# Dysregulation of miRNA–mRNA expression in fetal growth restriction in a caloric restricted mouse model

**DOI:** 10.1038/s41598-024-56155-6

**Published:** 2024-03-07

**Authors:** Lauren T. Gallagher, James Bardill, Carmen C. Sucharov, Clyde J. Wright, Anis Karimpour-Fard, Miguel Zarate, Courtney Breckenfelder, Kenneth W. Liechty, S. Christopher Derderian

**Affiliations:** 1 Department of Surgery, University of Colorado School of Medicine, Aurora, CO 80045 USA; 2 Laboratory for Fetal and Regenerative Biology, Department of Surgery, University of Colorado Denver School of Medicine, Aurora, CO 80045 USA; 3 Division of Cardiology, Department of Medicine, University of Colorado School of Medicine, Aurora, CO 80045 USA; 4 Section of Neonatology, Department of Pediatrics, University of Colorado School of Medicine, Aurora, CO 80045 USA; 5 Department of Biomedical Informatics, University of Colorado School of Medicine, Aurora, CO 80045 USA; 6 Division of Pediatric Surgery, University of Arizona College of Medicine, Tucson, AZ 85721 USA; 7 Colorado Fetal Care Center, Children’s Hospital Colorado, University of Colorado, 13123 E 16th Ave, Aurora, CO 80045 USA; 8 Division of Pediatric Surgery, University of Colorado School of Medicine and Children’s Hospital Colorado, Aurora, CO 80045 USA

**Keywords:** Angiogenesis, Disease model, Intrauterine growth

## Abstract

Fetal growth restriction (FGR) is associated with aberrant placentation and accounts for a significant proportion of perinatal deaths. microRNAs have been shown to be dysregulated in FGR. The purpose of this study was to determine microRNA-regulated molecular pathways altered using a caloric restricted mouse model of FGR. Pregnant mice were subjected to a 50% caloric restricted diet beginning at E9. At E18.5, RNA sequencing of placental tissue was performed to identify differences in gene expression between caloric restricted and control placentas. Significant differences in gene expression between caloric restricted and control placentas were observed in 228 of the 1546 (14.7%) microRNAs. Functional analysis of microRNA–mRNA interactions demonstrated enrichment of several biological pathways with oxidative stress, apoptosis, and autophagy pathways upregulated and angiogenesis and signal transduction pathways downregulated. Ingenuity pathway analysis also suggested that ID1 signaling, a pathway integral for trophoblast differentiation, is also dysregulated in caloric restricted placentas. Thus, a maternal caloric restriction mouse model of FGR results in aberrant microRNA-regulated molecular pathways associated with angiogenesis, oxidative stress, signal transduction, apoptosis, and cell differentiation. As several of these pathways are dysregulated in human FGR, our findings suggest that this model may provide an excellent means to study placental microRNA derangements seen in FGR.

## Introduction

Fetal growth restriction (FGR) refers to poor in utero growth and is defined by a fetal weight below the 10th percentile for a given gestational age^1^. The clinical implications are substantial as FGR affects 3–10% of pregnancies and accounts for nearly 50% of stillbirths^2,3^. Aside from the associated perinatal morbidity and mortality, FGR is a risk factor for multiple life-long comorbidities to include chronic inflammatory diseases, obesity, diabetes mellitus type II, and cardiovascular diseases^4,5^.

Despite multiple maternal and fetal risk factors, placental pathology is a hallmark finding in many cases of FGR^6,7^. In normal placentation, trophoblasts orchestrate the invasion and maturation of an intricate vascular network within the villous placenta^8^. This network is responsible for transporting nutrients, oxygen, and waste products to and from the fetus. In FGR, however, angiogenesis within the villous placenta is often impaired^9^. Consequently, insufficient oxygen and nutrients are delivered to the fetus, resulting in poor fetal growth^10^.

Multiple models to study FGR have been proposed. In animals, these include an ovine hyperthermia model, rodent uterine artery ligation model, maternal hypoxia model, and maternal nutrient-restriction model^11–15^. In vitro, the metabolic stress associated with FGR has been modeled by exposing trophoblast cell lines to either nutrient-restricted media or hypoxic conditions^16,17^. Although no model perfectly recreates FGR, nutrient-restricted models provide a holistic approach with similar sequelae observed in the human condition^15,17^.

microRNAs (miRNAs) are short non-coding RNAs composed of approximately 18–22 nucleotides. They regulate gene expression at the post-transcriptional level by targeting and binding the 3′ untranslated region (UTR) of specific messenger RNAs (mRNAs)^18^. This interaction, in turn, promotes mRNA degradation or suppression of mRNA translation. Thus, miRNAs largely repress protein production. In recent years, it has been shown that trophoblast proliferation, differentiation, and invasion are regulated by miRNAs within the placenta^19–23^. Furthermore, a subset of miRNAs is dysregulated in FGR^24,25^.

Previous studies sequencing human placental tissue effected by FGR and preeclampsia have identified multiple miRNA-regulated pathways associated with placental pathology. These include miR-193b-5p, miR-193-3p, miR-210-3p, miR-365a-3p, miR365b-3p, and miR-520a-3p which have been shown to regulated molecular pathways including inflammation, cell adhesion, cell migration, cell proliferation, and cell communication^26^. A similar study analyzing human growth restricted placentas also reported that miR193b-3p was differentially expressed but also identified other dysregulated miRNAs to include miR-379-3p and miR-335-3p which regulate pathways associated with inflammation and insulin-IGF^27^.

The aim of this analysis was to bridge the translational gap between human and mouse FGR research. We evaluated miRNA expression in a caloric restricted mouse model of FGR, and, using an existing mRNA-sequencing (mRNA-seq) dataset, we analyzed candidate pathways targeted by the identified miRNAs. Characterizing miRNA–mRNA interaction altered in unique disease processes such as FGR may identify culpable molecular pathways associated with disease pathogenesis. In this study, bioinformatics, including PANTHER and Ingenuity Pathways Analysis (IPA), are used to analyze miRNA–mRNA interactions and identify molecular mechanism associated with FGR.

## Materials and methods

### FGR mouse model

After University of Colorado IACUC approval, mice were housed in accordance with Guide for the Care and Use of Laboratory for Animals protocol^28^. Timed matings were performed and vaginal plugs were screened for daily. Pregnant C57BL/6 mice were provided ad libitum access to food between E1–E8. From E9-E18, dams received either a 50% caloric restricted diet (FGR) or continued ad libitum access (controls) as previously described^15^. Mouse placentas were harvested from three different dams within the caloric restricted and control groups, snap frozen in liquid nitrogen, and stored at − 80 °C until analysis. No specific placental structures were dissected and studied separately. This study is reported in accordance with ARRIVE guidelines.

### miRNA and mRNA isolation

RNA was extracted using a column-based extraction technique. In brief, tissue preparation included adding lysis buffer to 50 mg of placental tissue and homogenization with an Omni tissue homogenizer (Omni International, Kennesaw, GA, USA). Total cellular miRNA was purified using the Qiagen miRNeasy Mini Kit (Qiagen, Valencia, CA, USA) and total cellular mRNA was extracted using Qiagen RNeasy Pure mRNA Bead Kit (Qiagen, Valencia, CA, USA) both per manufacturer's protocol. After extraction of both miRNA and mRNA, the Qiagen RNeasy MinElute Cleanup Kit (Qiagen, Valencia, CA, USA) was used to remove any contaminating DNA and concentrate samples. RNA concentration was measured using the NanoDrop 2000c spectrophotometer (Thermofisher Scientific, Waltham, MA, USA). Quality and quantity of total RNA were analyzed using Bioanalyzer 2100 (Agilent, CA, USA), and a RIN number > 7.0 confirmed sample integrity.

### miRNA library preparation

Following miRNA extraction, 1 µg of total RNA was used to prepare small RNA libraries according to TruSeq Small RNA Sample Prep Kits (Illumina, San Diego, USA) protocol. Single-end sequencing 50 bp was performed on an Illumina Hiseq 2500 (Hangzhou, China).

### RT-qPCR

Placental samples used for RT-qPCR were from a different set of fetal growth restricted and control animals to increase the validity of our results. Following RNA extraction, cDNA was synthesized, and relative mRNA and miRNA levels were evaluated by quantitative RT-qPCR using exon spanning primers. Quantification was performed using the cycle threshold (ΔΔCt) method. Primers for miRNAs as well as mRNAs of interest were used to compare levels of expression between caloric restricted and control placentas.

### Bioinformatics analysis

RNA-seq was performed by LC Sciences (Houston, Texas, USA). First, ACGT101-miR was utilized to remove adapter dimers, junk, low complexity, common RNA families (rRNA, tRNA, snRNA, snoRNA) and repeats. Unique sequences with length in 18–26 nucleotide were then mapped to *Mus* *musculus* precursors in miRBase 22.0 by BLAST search to identify known miRNAs and novel 3p- and 5p-derived miRNAs. Length variation at both 3′ and 5′ ends and one mismatch inside of the sequence were allowed in the alignment. The unique sequences mapping to *Mus* *musculus* mature miRNAs in hairpin arms were identified as known miRNAs. The unique sequences mapping to the other arm of known *Mus* *musculus* precursor hairpin opposite to the annotated mature miRNA-containing arm were considered novel 5p- or 3p derived miRNA candidates. The unmapped sequences were BLASTed against the specific genomes, and the hairpin RNA structures containing sequences were predicated from the flank 80 nucleotide sequences using RNAfold software (http://rna.tbi.univie.ac.at/cgi-bin/RNAfold.cgi).

### Analysis of differential expressed miRNAs

Differential expression of miRNAs based on normalized deep-sequencing counts was analyzed by selectively using Fisher exact test, Chi-squared test, Student *t* test, or ANOVA based on the experiments design. The significance threshold was set to be 0.01 and 0.05 in each test.

### Prediction of target genes of miRNAs

To predict the genes targeted by the most abundant miRNAs, two computational target prediction algorithms (TargetScan 50 and Miranda 3.3a) were used to identify miRNA binding sites. Finally, the data predicted by both algorithms were combined and the overlaps were calculated. The GO terms and KEGG Pathway of these most abundant miRNAs, miRNA targets were also annotated.

The top 100 miRNAs based on count value from caloric restricted samples were identified. The log 2 normalized miRNA counts were compared between caloric restricted and normal placentas. Next, these top 100 miRNAs were used to identify predicted mRNA targets. miRNA targets were downloaded from TargetScan (http://www.targetscan.org/) release 8.0, September 2021.

miRNA–mRNA prediction analysis was performed using previously published mRNA sequencing data in caloric restricted mouse models^29^. Only mRNA targets with significant differences between fetal growth restricted and control placentas by mRNA-seq were used for further analysis. Functional enrichment analysis was performed by the machine learning tools, PANTHER and IPA, to identify potential targets of the differentially expressed miRNAs.

### Statistical analysis

Spearman’s correlation analysis was used to identify correlations between miRNAs and mRNA targets by ranking the expression values of miRNAs and mRNAs within datasets and calculating the correlation based on these ranks in order to discern potential regulatory associations^30^. One proportion test was performed for each of the miRNAs as we previously described. The predicted targets that were significant by one proportion test were used for IPA. The one proportion test was calculated by significantly inversely correlated targets subtracted from total significantly correlated targets over the total significantly correlated mRNA targets (https://www.medcalc.org/calc/test_one_proportion.php). Statistical analyses are performed using Prism GraphPad version 6 (GraphPad Software, Inc., La Jolla, CA). The alpha value < 0.05 was considered statistically significant.

## Results

### Caloric restricted mouse model

Litter size, placental weights, and fetal weights were determined at E18.5. No significant differences in litter size were appreciated between groups. The average litter size for the control group was 7 ± 2.6 and the average litter size for the caloric restriction group was 8.3 ± 0.6 (p = 0.44). Both fetal and placental weights were significantly lower in the caloric restriction group compared to the control group. The mean fetal weight at E18.5 for the control group was 0.86 ± 0.21 g compared to 0.45 ± 0.03 for the caloric restriction group (p = 0.0001, n ≥ 21 from 3 pregnancies). The mean placental weight at E18.5 for the control group was 0.11 ± 0.01 g compared to 0.07 ± 0.02 for the caloric restriction group (p = 0.0001, n ≥ 21 from 3 pregnancies).

### Differentially expressed miRNAs

**Figure 1:**
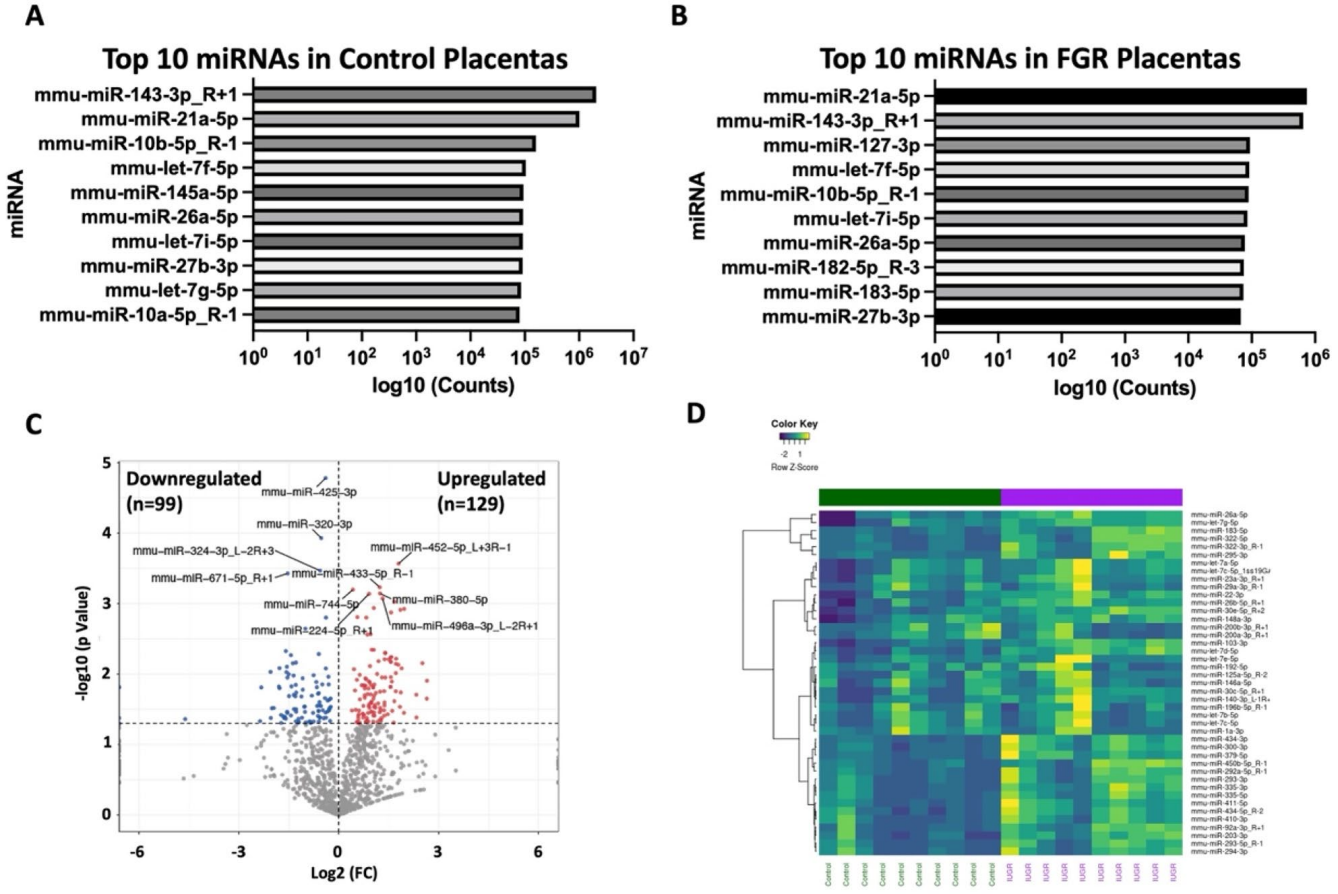
microRNA sequencing analysis. microRNAs with the highest level of expression within control placentas (**A**). microRNAs with the highest level of expression within control placentas (**B**). Volcano plot illustrating significantly downregulated microRNAs within fetal growth restricted compared to control placentas (left upper quadrant) and significantly upregulated microRNAs within fetal growth restricted compared to control placentas (right upper quadrant, **C**). Heat map representing the 42 top microRNAs based on counts significantly different between fetal growth restricted and control placentas (**D**).

We first compared expression profiles of miRNAs between fetal growth restricted and control placentas using RNA-sequencing (RNA-seq). In total, 1546 unique miRNAs were identified in control and caloric restriction placentas (Supplemental Table 1). The top 10 most highly expressed miRNAs are listed in Fig. 1A,B for control and caloric restriction placentas, respectively.

Following normalization using edgeR, further analysis of the 1546 unique miRNA identified 228 (14.7%) which had significantly different gene expression between the groups (p < 0.05, Supplemental Table 2). Of these, 129 (56.6%) miRNAs were upregulated and 99 (43.4%) miRNAs were downregulated (Fig. 1C). As the intent of this analysis was to identify differences in miRNA regulated molecular pathways between control and caloric restriction placentas, we focused on the top 100 miRNAs with the highest level of gene expression within fetal growth restricted placentas. Notably, 42 of the differentially expressed genes were found within the top 100 counts of the caloric restricted placentas, illustrated in the heatmap in Fig. 1D and tabular formation in Supplemental Table 3.

### Validation of sequencing data with RT-qPCR

**Figure 2:**
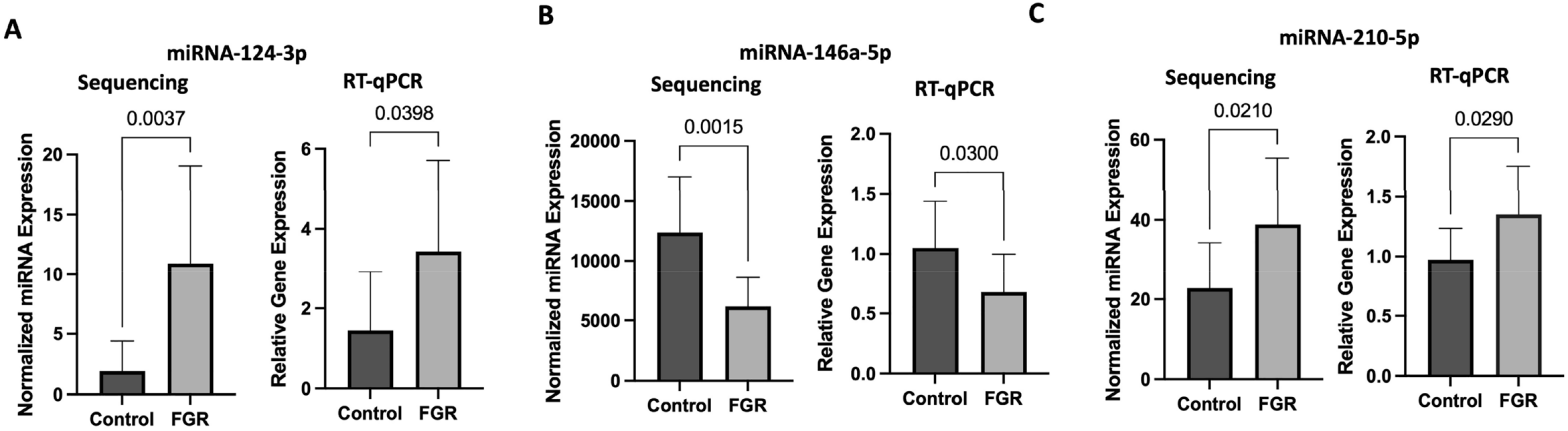
microRNA RT-qPCR and RNA-sequencing comparison. microRNA comparative analysis performed by RT-qPCR and RNA-seq between fetal growth restricted and control placentas among microRNAs found to have highly significantly different level of gene expression. N = 10/group and p-values were generated by Student *t* test. Data expressed as means with error bars representing standard deviations.

miRNA-Seq data was next confirmed by RT-qPCR. We selected three miRNAs that were highly expressed and had differential expression between caloric restriction and control placentas based on one proportion test (miR-124-3p, miR-146a-5p, and miR-210-5p). In addition, these miRNAs have known roles in regulating angiogenic pathways^31–34^. miRNA relative gene expression was normalized to miR-872-5p which had a stable count value across samples. Results from RT-qPCR corroborated RNA-Seq results and are presented in Fig. 2.

### Ingenuity pathways analysis

Next, IPA was utilized to predict putative target genes of dysregulated miRNAs identified within caloric restriction placentas. To so identify potential targets, we used a previously published mRNA-Seq dataset from placenta isolated from control and caloric restriction pregnancies generated using a similar mouse model^29^. This dataset reported 667 FPKM > 1 differentially expressed mRNA genes in placentas from control and caloric restriction. After selecting the 42 significantly different miRNAs that were found in the top 100 expressed miRNA in fetal growth restricted placentas within our dataset, we performed a Spearman correlation with one proportion test. Based on this analysis, 24 miRNAs were significantly dysregulated. Of them, 16 had inversely significant mRNA targets and were therefore used for functional analysis.

Among these 16 inversely related miRNA–mRNA interactions, 5 miRNAs were upregulated and 11 were downregulated (Table 1). Using these 16 miRNAs, IPA identified 276 enriched signaling pathways. The top pathways are described in Table 2 and a full list reported in Supplemental Table 4. Specific pathways involved in cell signaling (epithelial adherens junction signaling), angiogenesis (ID1 and TSP1), and mitogen-activated protein kinase (MAPK) pathway (ERK5) were enriched.

**Table 1:** Top dysregulated miRNAs.

miRNA	p-value	Confidence interval	Inversely related	FGR miRNA Expression
mmu-miR-410-3p	0.0001	29.04–96.33	Yes	↑
mmu-let-7e-5p	0.0001	48.80–90.85	Yes	↓
mmu-miR-103-3p	0.0001	19.41–99.37	Yes	↓
mmu-miR-125a-5p	0.0001	51.59–97.91	Yes	↓
mmu-miR-200b-3p	0.0001	44.33–82.79	Yes	↓
mmu-miR-30c-5p	0.0001	51.91–95.67	Yes	↓
mmu-miR-200a-3p	0.0003	45.13–86.14	Yes	↓
mmu-miR-146a-5p	0.0040	47.35–99.68	Yes	↓
mmu-miR-434-5p	0.0052	14.66–94.73	Yes	↑
mmu-miR-294-3p	0.0001	15.81–100.00	Yes	↑
mmu-miR-300-3p	0.0001	28.86–75.55	Yes	↑
mmu-miR-1a-3p	0.0001	19.41–99.37	Yes	↓
mmu-miR-23a-3p	0.0001	18.41–90.10	Yes	↓
mmu-miR-26b-5p	0.0001	54.43–93.95	Yes	↓
mmu-miR-29a-3p	0.0001	18.41–90.10	Yes	↓
mmu-miR-379-5p	0.0143	15.81–100.00	Yes	↑
mmu-miR-322-3p	0.0001	19.63–51.35	No	↑
mmu-let-7a-5p	0.0006	5.27–85.34	No	↓
mmu-miR-335-3p	0.0001	23.14–50.20	No	↑
mmu-miR-434-3p	0.0001	28.11–63.65	No	↑
mmu-miR-30e-5p	0.0230	28.67–68.05	No	↓
mmu-miR-26a-5p	0.0365	4.33–77.72	No	↓
mmu-let-7c-5p	0.0001	11.81–88.19	=	↓
mmu-miR-22-3p	0.0001	39.7–100.00	=	↓

microRNAs found to be significantly dysregulated based on Spearman correlation with one proportion test. = indicates the same number of negatively and positively related mRNA gene targets. *FGR* fetal growth restriction.

**Table 2:** Dysregulated ingenuity canonical pathways.

Ingenuity canonical pathways	− log(p-value)
Epithelial adherens junction signaling	2.93
Sperm motility	2.73
Role of tissue factor in cancer	2.56
ID1 signaling pathway	2.47
ERK5 signaling	2.21
Inhibition of angiogenesis by TSP1	1.9
Tight junction signaling	1.9
Glutamate degradation II	1.83
*N*-acetylglucosamine degradation I	1.83
Aspartate biosynthesis	1.83
Docosahexaenoic acid (DHA) signaling	1.81
Pyrimidine ribonucleotides interconversion	1.81
Natural killer cell signaling	1.76
Synaptic long-term depression	1.76
Pyrimidine ribonucleotides de novo biosynthesis	1.75
Ephrin receptor signaling	1.73
3-Phosphoinositide biosynthesis	1.71
l-Cysteine degradation I	1.7
*N*-acetylglucosamine degradation II	1.7
ERK/MAPK signaling	1.64
Ephrin A signaling	1.64

Top ingenuity pathway analysis-enriched canonical pathways.

### Validation with RT-qPCR

**Figure 3:**
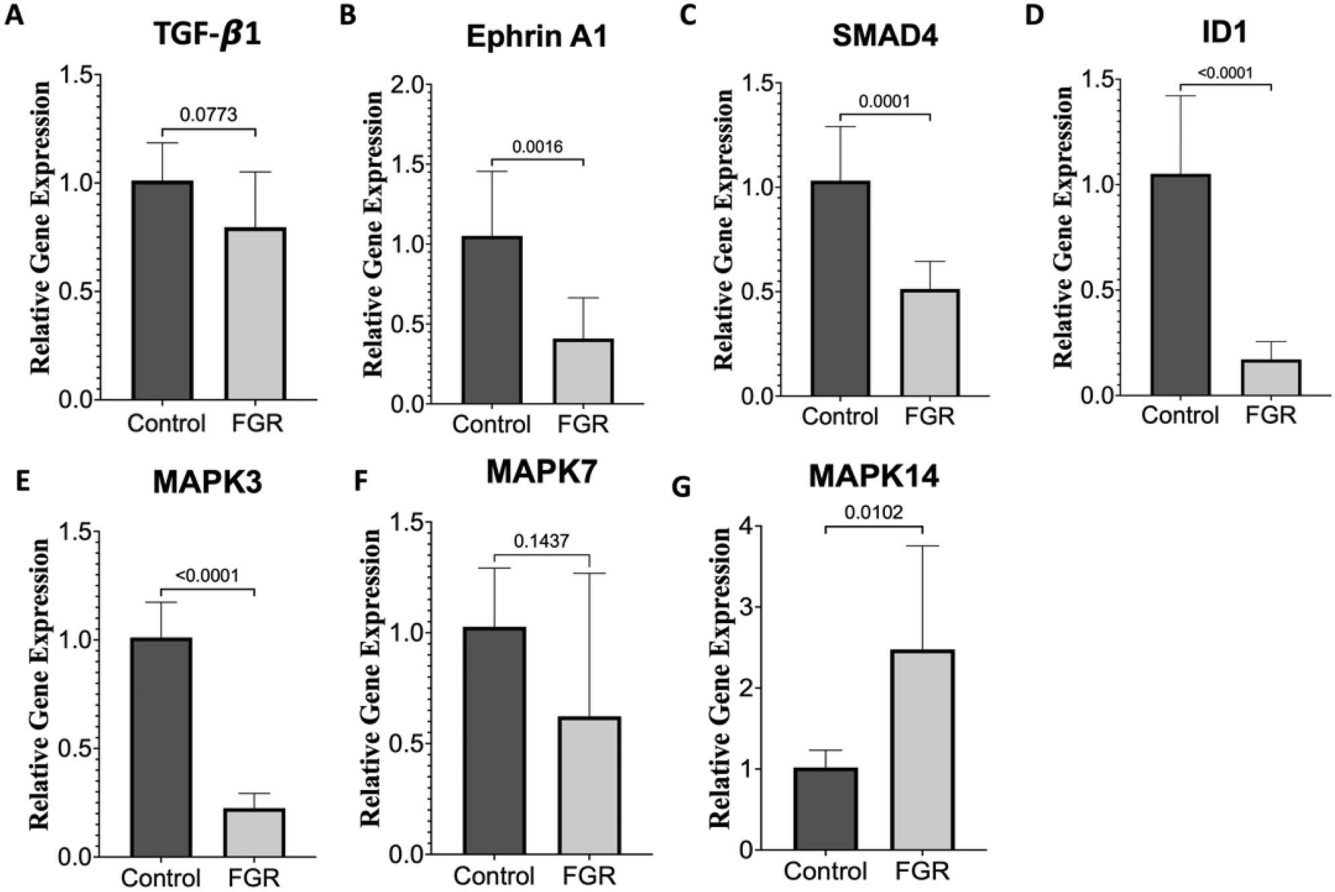
RT-qPCR analysis of predicted dysregulated pathways associated with fetal growth restriction. mRNA relative gene expression of proteins predicted to be dysregulated based upon IPA. N = 10/group and p-values were generated by Student *t* test. Data expressed as means with error bars representing standard deviations.

PANTHER and IPA both suggested an upregulation of MAPK pathways and downregulation of pathways associated with angiogenesis. To further validate our predictive models, we chose mRNA targets involved in these pathways as well as other identified pathways suggested to be dysregulated in FGR. Using RT-qPCR, we found that the relative gene expression of targeted mRNAs correlated with our PANTHER and IPA results with upregulation of the p38 MAP kinase pathway (MAPK14 pathway) and the downregulation of SMAD4, ERK1 (MAPK3), ephrin signaling, ID1 signaling and MAPK3 (Fig. 3). TGF-beta 1 and MAPK7 trended in similar directions but did not reach statistical significance.

### Functional enrichment analysis

**Figure 4:**
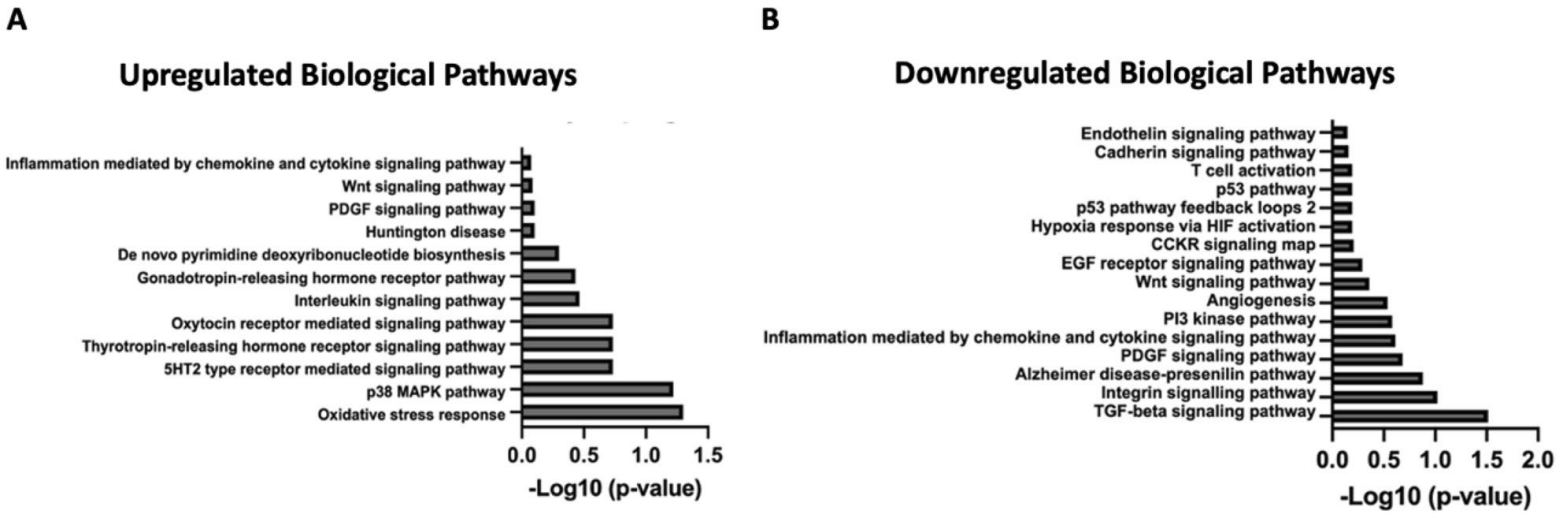
Biological pathways predicted to be dysregulated in fetal growth restriction. Canonical pathways predicted to be upregulated (**A**) and downregulated (**B**) in fetal growth restricted E18.5 placentas by Ingenuity Pathway Analysis.

To further identify differential effects by miRNAs specific to FGR, we performed a functional analysis using PANTHER database. To do so, we input the 24 significantly dysregulated miRNAs as well as the 667 mRNA targets into PANTHER which demonstrated 28 biological pathways that were enriched. Of these, 12 pathways were positively impacted and 16 were negatively impacted (Fig. 4). Of note, caloric restricted placentas had miRNAs that both upregulated and downregulated the Wnt pathway. This phenomenon results from complex gene networking; however, the balance of this gene overlap resulted in a an overall downregulatory effect. Supplemental Table 5 list each of the 24 dysregulated miRNAs and their potential mRNA targets. The most pronounced differences in gene expression were pathways associated with oxidative stress, extracellular signaling, and autophagy (p38 MAPK) which were upregulated. Pathways associated with cell proliferation (TGF-beta) and integrin signaling had the most pronounced reduction in gene expression within caloric restricted placentas. Angiogenesis, a mechanism thought to be integrally involved with the pathogenesis of FGR, was also downregulated compared to controls.

## Discussion

Fetal growth restriction has been a major source of perinatal morbidity and mortality for decades with few advancements in care. Despite the high prevalence, little is known about the molecular processes that drive its pathogenesis. Herein, we use a caloric restricted mouse model of FGR to identify aberrant miRNAs and their targeted pathways. Using functional enrichment analysis, we found that the metabolic stress associated with maternal malnutrition results in decreased cell growth, signal transduction, and angiogenesis as well as increased oxidative stress, apoptosis and autophagy within the placenta. While several of these pathways have independently been described to be associated with FGR, our analysis identifies many overlapping pathways, highlighting the complexity of the pathogenesis of FGR. It is critical to note that the relationship between caloric restriction and molecular signaling pathways is complex and multifaceted with little data within the literature. The effects of malnutrition on specific signaling cascades can vary depending on factors such as the type of malnutrition, the developmental stage, and the tissue or organ involved.

Two pathways highlighted in this work (Ephrin-A1 and ID1) have not yet been described in relation to maternal diet restriction or FGR. On the other hand, other animal models of maternal caloric restriction have shown similar pathway derangements to those shown here, specifically p38 MAPK. For instance, periconceptional nutrient restriction in pregnant sheep has been shown to alter MAPK/ERK1/2 and PI3K/Akt growth signaling pathways and vascularity in the placenta^35^.

It is important to highlight additional pathways that have been reported in FGR and diet restricted models for FGR. Maternal caloric restriction during pregnancy has been shown to be associated with alterations in molecular signaling pathways that play critical roles in FGR^36^. One major pathway affected is the insulin-like growth factor (IGF) pathway, critical to the regulation of cell proliferation and differentiation during development^37^. The mechanistic target of rapamycin (mTOR) pathway, involved in nutrient sensing and cellular growth, may also be influenced by caloric restriction, impacting cellular processes crucial for fetal development. Nutrient transporters are of particular interest in FGR and maternal nutrient deficiency. mTOR, which plays an important role in amino acid transportation, has been found to be downregulated in human FGR placentas^38^. In a pregnant primate study, maternal nutrient restriction caused inhibition of mTOR, ERK1/2, and insulin/IGF-1 signaling resulting in down-regulation of placental nutrient transporters. The reduction in nutrient transporters contributes to reduced fetal nutrient availability and FGR^39^. Another study showed reduction in placental transport amino acids in pregnant rats given low protein diets in an FGR model as a result of altered endocrine signaling, specifically reduced leptin, insulin/IGF-1, STAT3, and mTOR signaling^40–42^. Furthermore, a recent study showed alterations in nutrient transporter expression in FGR and preeclampsia, specifically the amino acid transporters SCL7A7 and SLC38A5^43^. Ongoing research continues to unravel the dynamic interplay of these molecular pathways, providing valuable insights into the consequences of maternal caloric restriction on fetal growth and development.

We provide data linking placental miRNA expression and dysregulated mRNA expression for targets relevant to the pathogenesis of FGR. Trophoblasts, which are the first cell line to develop from the fertilized egg, acquire a unique invasive phenotype during placentation^44^. This cell population migrates into the decidua, promoting spiral artery remodeling via interactions with endothelial cells. Aberrations within this invasive process have been linked to pregnancy complications such as preeclampsia and FGR^45^. Ephrin-A1 is a cell surface ligand that binds the Eph receptor. Although ephrin-A1 binding regulates many developmental processes to include neural and epithelial cell migration, it plays an important role in angiogenesis^46^. Within the placenta, ephrin-A1 is exclusively found within the extravillous trophoblast lineage and promotes placental incorporation into the uterine wall as well as trophoblast invasion into the maternal spiral arteries^47^. Our data suggests that both the ephrin receptor signaling pathway as well as gene expression of the ephrin-A1 ligand are downregulated in our caloric restriction mouse model. In addition, our functional analysis also suggest that biological pathways associated with angiogenesis are downregulated in our caloric restricted model. ID1 was predicted to be dysregulated within caloric restriction placental tissue. ID1, which is a DNA-binding protein inhibitor, has been shown to promote extravillous trophoblast invasion and differentiation and play an integral role in endothelial-like tube formation associated with angiogenesis^48,49^. These results synergistically support the current thought that aberrant placental angiogenesis is central to the pathogenesis of FGR and that miRNAs that regulate ephrin-A1 expression may be a driver of impaired angiogenesis in FGR.

In addition to dysregulated angiogenic pathways, we also found miRNA-regulated pathways associated with increased apoptosis and autophagy to be associated with caloric restriction. Our functional analysis using PANTHER demonstrated a significant upregulation of the p38 MAPK pathways. In addition, gene expression levels of MAPK14, a member of the p38 MAPK family, was also upregulated in caloric restricted placentas. Taken together, we postulate that impaired angiogenesis leads to a relative hypoxic state for both the placenta and fetus. As oxidative stress is a stimulus for the p38 MAPK pathway, upregulation of apoptosis and autophagy ensues^50^. Thus, our findings lead us to hypothesize that the increased cell turnover is responsive rather than causative in FGR.

The role of miRNAs in FGR remain elusive. Normal placentation is dependent upon miRNAs to regulate trophoblast proliferation, differentiation, and invasion^19–23^. Experiments in which mice lack the Dicer protein necessary to cleave pre-miRNA to mature miRNA result in fetal demise, an outcome that is preceded by impaired angiogenesis within the yolk sac and embryo^51^. This study highlights the importance of both miRNAs and angiogenesis in normal placental development. In a systematic review coalescing reports of microRNA signatures associate with FGR, Kochhar et al. identified 21 relevant studies. In more than one study, miR-210-5p and miR-424 were found to be upregulated and miR-518b, miR-519d and miR-221-3p were found to be downregulated^52^. From our analysis, we only identified two of these miRNAs within our mouse samples (miR-210 and miR-221-3p). Like the findings in this review, we also found miR-210 to be upregulated and miR-221-3p to be downregulated. While the role of these miRNAs in FGR is not clearly defined, the congruency of our results suggest that our animal model provides a valuable tool to study FGR. A similar study conducted in maternal rats with caloric restriction also demonstrated the role of several miRNA on target genes in angiogenesis and extracellular matrix remodeling, further suggesting nutritional status can have effects on the mechanisms of vessel formation of the fetus^53^. It should, however, be noted that there is limited evidence in rodent studies demonstrating how malnutrition is a cause of maternal vascular malperfusion. While the nutrient-restriction model used in this study may not be a known cause of maternal vascular malperfusion, specific nutrient deprivation has been reported in the literature for its associations with pregnancy complications. For instance, iron deficiency in mice was associated with impaired arterial network formation in the placenta^54^. In humans, low maternal folate nutritional status is reported to be associated with an increased risk of placental maternal vascular malperfusion and preeclampsia^55^.

This study has several limitations. First, we chose a caloric restricted model of FGR rather than other models to include a mouse uterine artery ligation model and sheep hyperthermia model. We chose maternal malnutrition as it is both a significant risk factor for FGR and, arguably, it is more physiological than other models described. This model results in reduced fetal weight and has metabolic sequelae to include glucose intolerance, impaired cognitive function and chronic diseases such as hypertension and coronary artery disease in response to reduced calories compared to mice that receive adequate nutrition^14,56,57^. Nevertheless, studies to include additional modeling strategies such as maternal hypoxia would strengthen the validity of our results. Second, we did not sex match placental tissue for this study. As several reports suggest that levels of miRNA expression may be altered based on sex differences in FGR, future work will include a detailed analysis of miRNA alterations as they relate to sex. It is also important to recognize that caloric restriction was initiated at E9. While an earlier timepoint would have been ideal, we feared that it would result in failed implantation. In addition, human and mouse placental anatomy is somewhat different. For instance, in humans, trophoblasts invade much deeper into the uterine wall, and in mice, the labyrinth zone is more extensive^58^. However, given that the placental developmental timeline of the mouse parallels the first half of human placentation, we consider this model to be quite applicable to human disease^58^. While mouse models of nutrient restriction provide valuable insights into the effects of limited nutrient availability on fetal development, differences in gestation period, metabolic rate, placental morphology, and maternal–fetal interactions between mice and humans require careful consideration.

Finally, although most miRNAs and their targeted mRNAs are conserved between humans and mice, not all are, which was evident when we compared our results to that of a systematic review analyzing human placental tissue^52^. As such, miRNAs and molecular pathways altered in mice may be different than those altered in humans^59^. However, several canonical pathways reported to be altered in human studies were also altered in our mouse model, specifically angiogenic, oxidative stress, and cell signaling pathways. Given the pathways identified and their relevance to the pathogenesis of FGR in humans, it is reasonable to hypothesize that some if not all these targets are conserved.

Herein, we use a nutrient deficient mouse model of FGR induced by maternal caloric restriction to mimic the malnourished state associated with many cases of FGR^5^. Our findings provide a framework of molecular pathways altered in FGR. Further work to characterize specific miRNAs that contribute to the pathogenesis of FGR, specifically anti-angiogenic ones, may be useful to identify therapeutic targets for in utero therapy in order to mitigate or reverse the FGR phenotype.

### Supplementary Information

Supplementary Table 1.

Supplementary Table 2.

Supplementary Table 3.

Supplementary Table 4.

Supplementary Table 5.

## Data Availability

All data generated or analyzed during this study are included in this published article (and its Supplementary Information files).

## References

[1] American College of Obstetricians, Gynecologists’ Committee on Practice Bulletins-Obstetrics and the Society for Maternal Fetal Medicine (2019). ACOG Practice Bulletin No. 204: Fetal growth restriction. Obstet. Gynecol..

[2] Faraci M (2011). Fetal growth restriction: Current perspectives. J. Prenat. Med..

[3] Higashijima A (2013). Characterization of placenta-specific microRNAs in fetal growth restriction pregnancy. Prenat. Diagn..

[4] Hales CN, Barker DJ (2001). The thrifty phenotype hypothesis. Br. Med. Bull..

[5] Black RE (2013). Maternal and child undernutrition and overweight in low-income and middle-income countries. Lancet.

[6] Veerbeek JH (2014). Placental pathology in early intrauterine growth restriction associated with maternal hypertension. Placenta.

[7] Krebs C (1996). Intrauterine growth restriction with absent end-diastolic flow velocity in the umbilical artery is associated with maldevelopment of the placental terminal villous tree. Am. J. Obstet. Gynecol..

[8] Pijnenborg R, Vercruysse L, Hanssens M (2006). The uterine spiral arteries in human pregnancy: Facts and controversies. Placenta.

[9] Ahmed A, Perkins J (2000). Angiogenesis and intrauterine growth restriction. Baillieres Best Pract. Res. Clin. Obstet. Gynaecol..

[10] Ravikumar G (2019). Placental expression of angiogenesis-related genes and their receptors in IUGR pregnancies: correlation with fetoplacental and maternal parameters. J. Matern. Fetal Neonatal Med..

[11] Habli M, Jones H, Aronow B, Omar K, Crombleholme TM (2013). Recapitulation of characteristics of human placental vascular insufficiency in a novel mouse model. Placenta.

[12] Janot M, Cortes-Dubly ML, Rodriguez S, Huynh-Do U (2014). Bilateral uterine vessel ligation as a model of intrauterine growth restriction in mice. Reprod. Biol. Endocrinol..

[13] Morrison JL (2008). Sheep models of intrauterine growth restriction: Fetal adaptations and consequences. Clin. Exp. Pharmacol. Physiol..

[14] Radford BN, Han VKM (2019). Offspring from maternal nutrient restriction in mice show variations in adult glucose metabolism similar to human fetal growth restriction. J. Dev. Orig. Health Dis..

[15] Zarate MA (2021). The acute hepatic NF-kappaB-mediated proinflammatory response to endotoxemia is attenuated in intrauterine growth-restricted newborn mice. Front. Immunol..

[16] Li L, Huang X, He Z, Xiong Y, Fang Q (2019). miRNA-210-3p regulates trophoblast proliferation and invasiveness through fibroblast growth factor 1 in selective intrauterine growth restriction. J. Cell. Mol. Med..

[17] Thamotharan S (2017). Differential microRNA expression in human placentas of term intra-uterine growth restriction that regulates target genes mediating angiogenesis and amino acid transport. PLoS One.

[18] Mohr AM, Mott JL (2015). Overview of microRNA biology. Semin. Liver Dis..

[19] Doridot L (2014). miR-34a expression, epigenetic regulation, and function in human placental diseases. Epigenetics.

[20] Kumar P, Luo Y, Tudela C, Alexander JM, Mendelson CR (2013). The c-Myc-regulated microRNA-17~92 (miR-17~92) and miR-106a~363 clusters target hCYP19A1 and hGCM1 to inhibit human trophoblast differentiation. Mol. Cell. Biol..

[21] Dai Y (2012). MicroRNA-155 inhibits proliferation and migration of human extravillous trophoblast derived HTR-8/SVneo cells via down-regulating cyclin D1. Placenta.

[22] Gao WL (2012). The imprinted H19 gene regulates human placental trophoblast cell proliferation via encoding miR-675 that targets Nodal Modulator 1 (NOMO1). RNA Biol..

[23] Umemura K (2013). Roles of microRNA-34a in the pathogenesis of placenta accreta. J. Obstet. Gynaecol. Res..

[24] Yang M (2016). miR-15b-AGO2 play a critical role in HTR8/SVneo invasion and in a model of angiogenesis defects related to inflammation. Placenta.

[25] Sun LL, Li WD, Lei FR, Li XQ (2018). The regulatory role of microRNAs in angiogenesis-related diseases. J. Cell. Mol. Med..

[26] Ostling H, Kruse R, Helenius G, Lodefalk M (2019). Placental expression of microRNAs in infants born small for gestational age. Placenta.

[27] Awamleh Z, Gloor GB, Han VKM (2019). Placental microRNAs in pregnancies with early onset intrauterine growth restriction and preeclampsia: Potential impact on gene expression and pathophysiology. BMC Med. Genom..

[28] National Research Council Institute for Laboratory Animal Resources (1996). Guide for the Care and Use of Laboratory Animals.

[29] Chen PY (2013). Intrauterine calorie restriction affects placental DNA methylation and gene expression. Physiol. Genom..

[30] Hailu FT (2022). Integrated analysis of miRNA–mRNA interaction in pediatric dilated cardiomyopathy. Pediatr. Res..

[31] Cabello P (2023). miR-146a-5p promotes angiogenesis and confers trastuzumab resistance in HER2+ breast cancer. Cancers (Basel).

[32] Shi Y (2020). miR-124-3p regulates angiogenesis in peripheral arterial disease by targeting STAT3. Mol. Med. Rep..

[33] Shi Z (2014). MiR-124 governs glioma growth and angiogenesis and enhances chemosensitivity by targeting R-Ras and N-Ras. Neuro Oncol..

[34] Zeng L (2014). MicroRNA-210 overexpression induces angiogenesis and neurogenesis in the normal adult mouse brain. Gene Ther..

[35] Zhu MJ, Du M, Hess BW, Nathanielsz PW, Ford SP (2007). Periconceptional nutrient restriction in the ewe alters MAPK/ERK1/2 and PI3K/Akt growth signaling pathways and vascularity in the placentome. Placenta.

[36] Rachakatla A, Kalashikam RR (2022). Calorie Restriction-regulated molecular pathways and its impact on various age groups: An overview. DNA Cell Biol..

[37] Hawkes CP, Grimberg A (2015). Insulin-like growth factor-I is a marker for the nutritional state. Pediatr. Endocrinol. Rev..

[38] Roos S (2007). Mammalian target of rapamycin in the human placenta regulates leucine transport and is down-regulated in restricted fetal growth. J. Physiol..

[39] Kavitha JV (2014). Down-regulation of placental mTOR, insulin/IGF-I signaling, and nutrient transporters in response to maternal nutrient restriction in the baboon. FASEB J..

[40] Jansson N (2006). Down-regulation of placental transport of amino acids precedes the development of intrauterine growth restriction in rats fed a low protein diet. J. Physiol..

[41] Rosario FJ (2011). Maternal protein restriction in the rat inhibits placental insulin, mTOR, and STAT3 signaling and down-regulates placental amino acid transporters. Endocrinology.

[42] Sibley CP (2005). Placental phenotypes of intrauterine growth. Pediatr. Res..

[43] Huang X (2018). Identification of placental nutrient transporters associated with intrauterine growth restriction and pre-eclampsia. BMC Genom..

[44] Kingdom JC, Kaufmann P (1999). Oxygen and placental vascular development. Adv. Exp. Med. Biol..

[45] Brosens I, Puttemans P, Benagiano G (2019). Placental bed research: I. The placental bed: From spiral arteries remodeling to the great obstetrical syndromes. Am. J. Obstet. Gynecol..

[46] Ahmed Z, Bicknell R (2009). Angiogenic signalling pathways. Methods Mol. Biol..

[47] Goldman-Wohl D (2004). Eph and ephrin expression in normal placental development and preeclampsia. Placenta.

[48] Zhao HJ (2020). Bone morphogenetic protein 2 promotes human trophoblast cell invasion and endothelial-like tube formation through ID1-mediated upregulation of IGF binding protein-3. FASEB J..

[49] Renaud SJ (2015). OVO-like 1 regulates progenitor cell fate in human trophoblast development. Proc. Natl. Acad. Sci. USA.

[50] Canovas B, Nebreda AR (2021). Diversity and versatility of p38 kinase signalling in health and disease. Nat. Rev. Mol. Cell Biol..

[51] Yang WJ (2005). Dicer is required for embryonic angiogenesis during mouse development. J. Biol. Chem..

[52] Kochhar P, Vukku M, Rajashekhar R, Mukhopadhyay A (2022). microRNA signatures associated with fetal growth restriction: A systematic review. Eur. J. Clin. Nutr..

[53] Khorram O (2010). Effect of maternal undernutrition on vascular expression of micro and messenger RNA in newborn and aging offspring. Am. J. Physiol. Regul. Integr. Comp. Physiol..

[54] Kalisch-Smith JI (2021). Maternal iron deficiency perturbs embryonic cardiovascular development in mice. Nat. Commun..

[55] Chilukuri N (2022). Maternal folate status and placental vascular malperfusion: Findings from a high-risk US minority birth cohort. Placenta.

[56] Yanney M, Marlow N (2004). Paediatric consequences of fetal growth restriction. Semin. Fetal Neonatal Med..

[57] Barker DJ (1998). In utero programming of chronic disease. Clin. Sci. (Lond.).

[58] Soncin F (2018). Comparative analysis of mouse and human placentae across gestation reveals species-specific regulators of placental development. Development.

[59] Friedman RC, Farh KK, Burge CB, Bartel DP (2009). Most mammalian mRNAs are conserved targets of microRNAs. Genome Res..

